# Better lactate clearance associated with good neurologic outcome in survivors who treated with therapeutic hypothermia after out-of-hospital cardiac arrest

**DOI:** 10.1186/cc13090

**Published:** 2013-10-31

**Authors:** Tae Rim Lee, Mun Ju Kang, Won Chul Cha, Tae Gun Shin, Min Seob Sim, Ik Joon Jo, Keun Jeong Song, Yeon Kwon Jeong, Jun Hwi Cho

**Affiliations:** 1 Department of Emergency Medicine, Samsung Medical Center, Sungkyunkwan University School of Medicine, 135-710 Ilwon-Dong 50, Gangnam-Ku, Seoul, Republic of Korea; 2 Department of Emergency Medicine, College of Medicine, Kangwon National University, (135-710) 81 Irwon-Ro Gangnam-gu. Seoul, Chuncheon, Samsung Medical Center, Republic of Korea

## Abstract

**Introduction:**

Several methods have been proposed to evaluate neurological outcome in out-of-hospital cardiac arrest (OHCA) patients. Blood lactate has been recognized as a reliable prognostic marker for trauma, sepsis, or cardiac arrest. The objective of this study was to examine the association between initial lactate level or lactate clearance and neurologic outcome in OHCA survivors who were treated with therapeutic hypothermia.

**Methods:**

This retrospective cohort study included patients who underwent protocol-based 24-hour therapeutic hypothermia after OHCA between January 2010 and March 2012. Serum lactate levels were measured at the start of therapy (0 hours), and after 6 hours, 12 hours, 24 hours, 48 hours and 72 hours. The 6 hour and 12 hour lactate clearance were calculated afterwards. Patients’ neurologic outcome was assessed at one month after cardiac arrest; good neurological outcome was defined as Cerebral Performance Category one or two. The primary outcome was an association between initial lactate level and good neurologic outcome. The secondary outcome was an association between lactate clearance and good neurologic outcome in patients with initial lactate level >2.5 mmol/l.

**Results:**

Out of the 76 patients enrolled, 34 (44.7%) had a good neurologic outcome. The initial lactate level showed no significant difference between good and poor neurologic outcome groups (6.07 ±4 .09 mmol/L vs 7.13 ± 3.99 mmol/L, *P* = 0.42), However, lactate levels at 6 hours, 12 hours, 24 hours, and 48 hours in the good neurologic outcome group were lower than in the poor neurologic outcome group (3.81 ± 2.81 vs 6.00 ± 3.22 *P* <0.01, 2.95 ± 2.07 vs 5.00 ± 3.49 *P* <0.01, 2.17 ± 1.24 vs 3.86 ± 3.92 *P* <0.01, 1.57 ± 1.02 vs 2.21 ± 1.35 *P* = 0.03, respectively). The secondary analysis showed that the 6-hour and 12-hour lactate clearance was higher for good neurologic outcome patients (35.3 ± 34.6% vs 6.89 ± 47.4% *P* = 0.01, 54.5 ± 23.7% vs 25.6 ± 43.7% *P* <0.01, respectively). After adjusting for potential confounding variables, the 12-hour lactate clearance still showed a statistically significant difference (*P* = 0.02).

**Conclusion:**

The lactate clearance rate, and not the initial lactate level, was associated with neurological outcome in OHCA patients after therapeutic hypothermia.

## Introduction

Despite updates in cardiopulmonary resuscitation guidelines and advances in post-cardiac arrest care, there has been little improvement in survival rate and in hospital discharge with good neurologic outcome after out-of-hospital cardiac arrest (OHCA) [1,2]. Predicting survival or neurologic outcome after cardiac arrest still remains difficult; no reliable and accurate tools or parameters have been identified. There have been many methods proposed for evaluation and prediction of patients’ neurologic outcome and prognosis, such as evaluation of the Glasgow Coma Scale or brain stem function after return of spontaneous circulation (ROSC), or measurement of serum biomarkers [3,4] such as neuron-specific enolase, S100, serum ammonia [5] or serum lactate. Among these serum biomarkers, the blood lactate level has been suggested as a promising candidate for prognostic prediction in OHCA patients [6-10].

The main pathophysiology of post-cardiac arrest syndrome (PCAS) is a systemic ischemia/reperfusion response [11], which has much in common with severe sepsis or septic shock. Donnino and colleagues found that OHCA patients with better lactate clearance had decreased early and overall in-hospital mortality [12]. As in patients with septic shock, lactate can be used as a prognostic factor or predictor of outcome in OHCA patients. Kliegel and colleagues found that patients with lower lactate levels at admission and after 48 hours showed more favorable neurologic outcomes [13].

The 2010 America Heart Association guidelines recommend therapeutic hypothermia (TH) for comatose patients with ROSC after OHCA to protect the brain and other organs [14-17]. However, there has been no study of the relationship between lactate clearance and neurologic outcome in patients who underwent TH. The objectives of this study were to determine the association of lactate level and lactate clearance with neurologic outcome in OHCA survivors treated with TH.

## Materials and methods

We conducted protocol-based TH from January 2010 in patients who survived OHCA. On a retrospective basis, we collected data from a prospective database of OHCA patients from Samsung Medical Center, who had been treated with hypothermia between January 2010 and March 2012. This study was performed retrospectively using a coded database, so we did not need to collect informed consent about this study. The study protocol was reviewed and approved by the Institutional Review Board (file number 2012-06-063) in our hospital.

### Samsung medical center hypothermia protocol

We used TH for all OHCA patients after ROSC. Hypothermia induction was started by 4°C cold saline infusion (about 30 ml/kg) over 30 minutes. In addition, we used a commercial temperature regulation system that consisted of hydrogel pads (Arctic Sun Temperature Management System, Medivance, CO, USA). If the commercial device was not available, we used conventional methods such as a commercial cold blanket, 4°C cold saline bladder irrigation or traditional ice packs placed on the patient’s groin, armpits, and around the neck and head. The target temperature for induction was 33.5°C. TH was applied for 24 hours, with the patient’s core body temperature maintained at 33 ± 0.5°C. After the maintenance period, we rewarmed patients to 36.5°C at a rate of 0.15°C/hour on a hydrogel pad or at a rate lower than 0.25°C/hour on a cooling blanket. In the normothermic period after the temperature had reached 36.5°C, the patient’s core body temperature was maintained at less than 37.5°C until 72 hours after ROSC.

During TH, shivering was assessed using the Bedside Shivering Assessment Scale [18]. Shivering was controlled by intermittent intravenous pethidine (25 mg; maximum 100 mg/day) and intravenous magnesium sulfate (2 g intravenously, if serum magnesium <3 mg/dl) at Bedside Shivering Assessment Scale >2 (mild shivering). Neuromuscular blocking agents were used if shivering was not controlled with pethidine or magnesium, according to the decision of the attending physicians, and with electroencephalogram monitoring.

The hemodynamic optimization targets were as follows: central venous pressure ≥8 to 12 mmHg, mean arterial pressure ≥75 mmHg, peripheral oxygen saturation >94%, and partial pressure of carbon dioxide >40 mmHg. To attain these targets, dopamine was used as a first-line vasopressor (starting at 10 μg/kg/minute; maximum 30 μg/kg/minute), and norepinephrine (starting at 5 μg/minute; maximum 100mcg/minute) as a second choice. Thereafter, the use of vasopressin, an intra-arterial balloon pump or extracorporeal membrane oxygenation was determined by the attending physician.

Venous blood samples were collected at the following time points: at TH induction (0 hours), and after 6 hours, 12 hours, 24 hours, 48 hours, and 72 hours. The venous blood samples were stored in sodium fluoride/potassium oxalate tubes. These samples were immediately sent for laboratory testing and centrifuged at 3,500 rpm for 5 minutes to obtain the serum. Lactate levels were then measured by Modular DP analyzer® (Roche, Basel, Switzerland).

### Data collection and patient enrollment

The study protocol was reviewed and approved by the hospital Institutional Review Board. In the Samsung Medical Center TH database, data are recorded on standardized case forms following the Utstein style [19,20] along with the Sequential Organ Failure Assessment score from hypothermia induction. We defined organ failure as Sequential Organ Failure Assessment score >2 for the particular organ.

All adult OHCA patients (>18 years) who underwent TH after ROSC were enrolled into this study. The exclusion criteria for TH were as follows: the cause of arrest was sepsis, progression of malignancy, trauma, or hemorrhagic shock; expected survival was <3 months before cardiac arrest; the patient was capable of only limited self-care and was confined to a bed or chair for 50% or more of waking hours; the patient had a major operation (head, chest, abdomen and vascular) within 7 days; >12 hours had elapsed after ROSC; the patient had an unwitnessed arrest so the time of arrest could not be determined; the patient had an intracranial hemorrhage on brain noncontrast computerized tomography; or the patient was pregnant. Patients who were transferred to our hospital after hypothermia induction at different hospitals were excluded from this study because their blood samples for lactate measurement could not be obtained.

We divided patients into two groups to compare serial lactate levels according to neurologic outcome. Patients in the good neurologic outcome group had Cerebral Performance Category scores of 1 or 2 at 1 month after OHCA. Patients in the poor neurologic outcome group had Cerebral Performance Category scores from 3 to 5 at 1 month [21]. To compare lactate clearance, we identified patients with initial lactate levels >2.5 mmol/l. The 6-hour and 12-hour lactate clearance was calculated as follows:

6-hour lactate clearance (%) = (0-hour lactate – 6-hour lactate) / 0-hour lactate × 100

12-hour lactate clearance (%) = (0-hour lactate – 12-hour lactate) / 0-hour lactate × 100

### Statistical analysis

To compare the demographics of both groups, we used the chi-square test for nominal variables, the Student *t* test for continuous variables with a normal distribution, and the Wilcoxon rank-sum test for continuous variables without a normal distribution.

We performed univariate logistic regression for variables possibly associated with neurologic outcome. To evaluate the association of good neurologic outcome with lactate clearance level, we performed multivariate logistic regression analysis adjusting for variables with *P* <0.1 in the univariate analysis: age, no-flow time, automated external defibrillator shock, cause of arrest and initial rhythm.

Statistical analysis was performed using STATA 11.0 (StataCorp, Texas(TX), USA) and differences considered significant at *P* <0.05. In this study, we describe nominal data as numbers of cases and percentages, continuous variables with a normal distribution as mean ± standard deviation, and continuous variables without a normal distribution as median and interquartile range.

## Results

**Figure 1:**
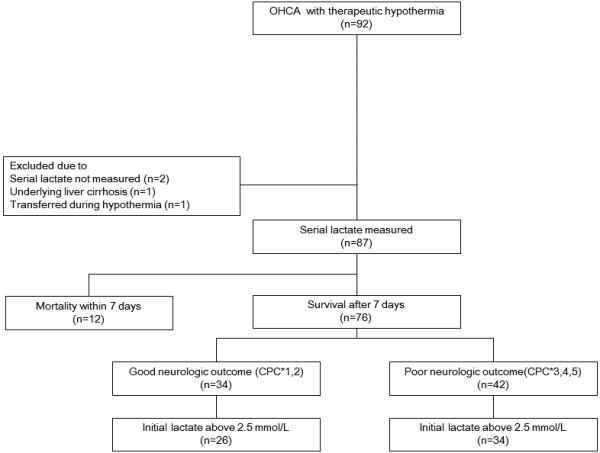
**Study enrollment.** CPC*, Cerebral Performance Category; OHCA, out-of-hospital cardiac arrest.

During the study period, a total of 92 OHCA patients underwent TH. One patient was excluded after transfer to another facility following TH with no proper neurologic outcome assessment. Two patients were excluded because of a lack of serial lactate measurements and one patient was excluded because of underlying liver cirrhosis. Twelve patients died within 7 days and were excluded from further analysis because they died before proper assessment for neurologic outcome. Finally, 76 patients (48 male, 28 female; mean age 51.7 years) were enrolled and were divided into two groups according to their neurologic outcome; 34 with a good neurologic outcome, and 42 with a poor neurologic outcome (Figure 1).

### Baseline characteristics of both groups

Table 1 presents the baseline characteristics of the enrolled patients. The demographic data did not show significant differences except for cause of arrest, bystander cardiopulmonary resuscitation, automated external defibrillator shock, no-flow time, and initial rhythms (*P* <0.01, *P* <0.01, *P* <0.01, *P* <0.01, and *P* <0.01, respectively). In the poor neurologic group, low-flow time was longer, and hypothermia induction time was shorter although the difference was not statistically significant (*P* = 0.43 and *P* = 0.53, respectively) (Table 1).

**Table 1:** Baseline characteristics of the study groups

	**Good neurologic group (*n*** **= 34)**	**Poor neurologic group (*n*** **= 42)**	***P*** **value**
Age	47.9 ± 15.9	54.9 ± 16.3	0.06
Gender male (%)	24 (70.6)	24 (57.1)	0.22
Weight (kg)	62.5 ± 13.6	61.7 ± 8.5	0.76
Height (cm)	167.1 ± 7.6	164.7 ± 8.6	0.22
Body mass index	22.2 ± 3.7	22.7 ± 2.1	0.51
Underlying disease			
Hypertension (%)	8 (23.5)	9 (21.4)	0.82
Diabetics (%)	4 (11.8)	6 (14.3)	0.74
Heart disease (%)	5 (14.7)	5 (11.9)	0.72
Chronic renal disease (%)	2 (5.8)	3 (7.1)	0.82
Malignancy (%)	4 (11.8)	3 (7.1)	0.49
Arrest cause (%)			<0.01
Cardiac	33 (97.1)	19 (45.2)	
Respiratory	1 (2.9)	22 (52.4)	
Other	0 (0)	1 (2.4)	
Bystander CPR (%)	18 (52.9)	9 (21.4)	<0.01
AED apply (%)	26 (76.4)	28 (66.7)	0.34
AED shock (%)	23 (67.7)	10 (23.8)	<0.01
No-flow time (minutes)	3.5 ± 3.8	12.7 ± 9.5	<0.01
Low-flow time (minutes)	22.9 ± 21.2	26.2 ± 15.8	0.43
BLS time (minutes)	10.3 ± 7.8	12.7 ± 9.2	0.22
ACLS time (minutes)	12.5 ± 19.2	13.4 ± 11.7	0.79
Initial rhythms (%)			<0.01
Ventricular fibrillation	24 (70.6)	9 (21.4)	
Pulseless electrical activity	8 (23.5)	16 (38.1)	
Asystole	2 (5.9)	17 (40.5)	
Hypothermia therapy induction time	194.6 ± 92.2	179 ± 113.5	0.53
Organ failure rate			
Heart (%)	24 (70.6)	26 (61.9)	0.42
Lung (%)	19 (55.8)	26 (61.9)	0.59
Liver (%)	0 (0.0)	0 (0.0)	–
Kidney (%)	6 (17.7)	2 (4.7)	0.06
Coagulation (%)	2 (6.1)	3 (8.1)	0.74
SOFA score (median)	6 (3 to 7)	5 (2 to 7)	0.66

Data presented as mean ± standard deviation, *n* (%) or median (interquartile range). AED, automated external defibrillator; BLS, basic life support; ACLS, advanced cardiac life support; CPR, cardiopulmonary resuscitation; SOFA, Sequential Organ Failure Assessment.

### Serial lactate level

**Figure 2:**
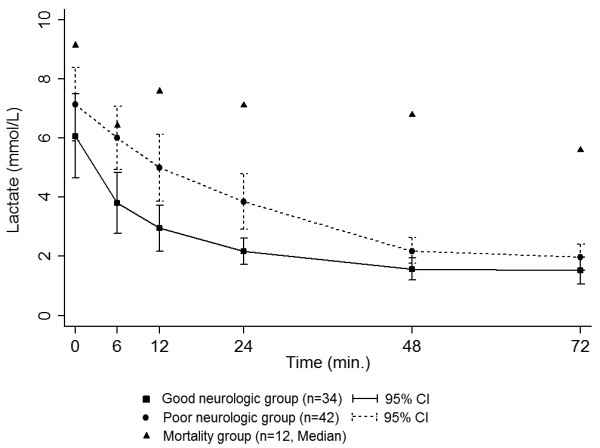
**Serial mean lactate levels for the two study groups and patients who died within 7 days.** CI, confidence interval.

There was no significant difference in the initial lactate level between the two groups (6.07 mmol/l in the good outcome group vs. 7.13 mmol/l in the poor outcome group, *P* = 0.42). However, lactate levels at 6 hours, 12 hours, 24 hours, and 48 hours in the good neurologic outcome group were significantly lower than those in the poor neurologic outcome group (3.81 vs. 6.00, *P* <0.01; 2.91 vs. 5.00, *P* <0.01; 2.17 vs. 3.86, *P* <0.01; and 1.57 vs. 2.21, *P* = 0.03, respectively). Lactate levels at 72 hours in both groups showed no statistically significant difference (1.52 vs. 1.97, *P* = 0.14) (Figure 2). In addition, for reference, we plotted the median lactate levels of a group of 12 patients who died within 7 days (Figure 2). These levels were higher than both groups of surviving patients in our study.

### Lactate clearance

**Figure 3:**
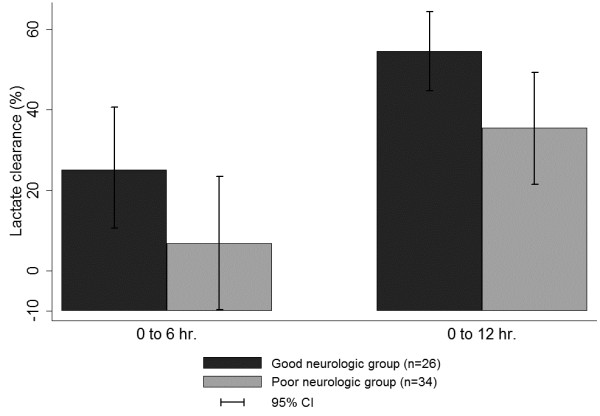
**Lactate clearance of subjects in the high lactate subgroup.** CI, confidence interval.

Patients with initial lactate above 2.5 mmol/l (26 (76.5%) of the good outcome group, 34 (81.0%) of the poor outcome group) were selected as the high lactate subgroup to determine the association between lactate clearance and neurologic outcome. The 6-hour and 12-hour lactate clearance in the good neurologic outcome group were higher than those in the poor neurologic outcome group (35.3% vs. 6.89%, *P* = 0.01; 54.5% vs. 25.6%, *P* <0.01), respectively) (Figure 3). In the multivariate regression model, 6-hour and 12-hour lactate clearance showed a significant association with good neurologic outcome after adjustment for confounders (odds ratio: 1.02, 95% confidence interval: 1.03 to 1.05, *P* = 0.03; and odds ratio: 1.03, 95% confidence interval: 1.00 to 1.07, *P* = 0.02) (Table 2).

**Table 2:** Multivariate logistic regression analysis of good neurologic outcome

	**Univariate**		**Multivariate with 6-hour lactate clearance**		**Multivariate with 12-hour lactate clearance**	
	**Unadjusted OR (95% CI)**	***P*** **value**	**Adjusted OR (95% CI)**	***P*** **value**	**Adjusted OR (95% CI)**	***P*** **value**
Age	0.97 (0.94 to 1.01)	0.14	0.93 (0.87 to 1.00)	0.05	0.92 (0.86 to 0.99)	0.04
No-flow time	0.80 (0.70 to 0.91)	<0.01	0.77 (0.61 to 0.95)	0.02	0.72 (0.57 to 0.91)	<0.01
Bystander CPR	4.04 (1.36 to 11.95)	0.01	0.34 (0.05 to 2.56)	0.29	0.21 (0.02 to 1.80)	0.15
AED shock	4.07 (1.41 to 11.68)	0.01	1.70 (0.25 to 12.92)	0.60	0.51 (0.07 to 3.58)	0.50
Initial rhythm (shockable rhythm)	5.47 (1.85 to 16.15)	<0.01	8.51 (1.03 to 70.26)	0.04	18.42 (1.93 to 175.32)	0.01
Arrest cause (cardiac)	26.6 (3.28 to 215.30)	<0.01	–	–	–	
6-hour lactate clearance	1.02 (1.00 to 1.03)	0.02	1.02 (1.03 to 1.05)	0.03	-–	
12-hour lactate clearance	1.03 (1.01 to 1.05)	<0.01	–	–	1.03 (1.00 to 1.07)	0.02

AED, automated external defibrillator; CI, confidence interval; CPR, cardiopulmonary resuscitation; OR, odds ratio.

## Discussion

The aim of our study was to examine the relationship between serum lactate and neurologic outcome of OHCA patients after TH. The initial lactate level showed no statistically significant difference but the lactate levels at 6 hours, 12 hours, 24 hours and 48 hours in the good neurologic outcome group were lower than in the poor neurologic outcome group. In addition, we found that effective early (6-hour and 12-hour) lactate clearance was associated with good neurologic outcome in OHCA patients treated with TH even after adjustment for potential confounding factors. There is no confirmed consensus regarding the cutoff point at which the lactate clearance is meaningful. Previous studies used values ranging from 2 to 3 mmol/l. We decided on 2.5 mmol/l as a reasonable cutoff value, because determination of lactate clearance in the normal range would not provide meaningful data. Sixteen patients were accordingly excluded. Three of these patients showed an increase in lactate level at 6 hours, and one had a good neurologic outcome (lactate increased to 3.6 mmol/l at 6 hours) while the other two had a poor neurologic outcome (lactate increased to 4.5 and 5.7 mmol/l, respectively, at 6 hours). However, subgroup analysis was not performed because of the small number of patients. Further investigation may be needed in patients whose initial lactate level is in the normal range but increases during the early phase of TH.

Lactate represents a useful marker of tissue hypoxia [22]. However, interpretation of single lactate measurements has several limitations because serum lactate levels reflect the difference between lactate production and elimination; an increased lactate level might indicate mechanisms other than cellular hypoxia [23]. Furthermore, a single measurement of serum lactate on admission has low accuracy in the prognosis of critically ill patients [24], but several studies have shown that serial lactate measurement might be a useful predictor of patient outcome in circulatory shock [22,25-27].

PCAS or post-resuscitation disease is a sepsis-like syndrome associated with ischemia/reperfusion injury and the inflammation cascade [11,28]. With or without profound arterial hypotension, an elevated serum lactate level can be a hallmark of impaired tissue perfusion in patients with severe sepsis or septic shock [29]. Thus, in a patient with severe sepsis or septic shock, better lactate clearance can be associated with decreased mortality and might arise from more rapid improvement in tissue perfusion. Most patients who die from PCAS have multiorgan failure, and their serum lactate level is increased. In critically ill patients, particularly those with sepsis, better lactate clearance has been associated with decreased mortality [23,24]. Similarly, in the PCAS patient, the lactate level and lactate clearance are associated with mortality and neurologic outcome. Donnino and colleagues found that early and effective lactate clearance is associated with decreased mortality in the PCAS patient [12]. In another study, persistent hyperlactemia at 48 hours seemed to predict mortality and an unfavorable neurologic outcome [13].

Our study focused on the association of lactate and its clearance with OHCA patients’ neurologic outcome but not their survival. We excluded patients who died within 7 days because they died before proper assessment of neurologic outcome was performed. However, these patients showed increased lactate levels during their hospital course, which we supposed might be caused by multiorgan failure leading to death. The number of deaths within 7 days was too small to determine the statistical significance of serial lactate levels, but the overall lactate level was higher and lactate clearance was lower in the nonsurvivors in our study.

In our study, the serum lactate level in both groups reached the normal level after 48 hours, and is different from a previous study that showed normalized lactate levels within 24 hours [13]. This may be due to decreased lactate metabolism and clearance during TH. These findings are different from a previous study of mild hypothermia therapy in patients with traumatic brain injury, in which the hypothermia group showed lower mean lactate levels and improved neurologic outcome [30]. As we could not compare lactate clearance between the TH group and a non-TH group because of a lack of OHCA patients without TH, the effect of TH on lactate clearance remains controversial [31].

In this study, we could not clearly identify how lactate clearance was associated with a good neurologic outcome of OHCA patients with TH. However, previous studies showed that hypothermia was associated with increased cerebral lactate clearance [32] and also with reduced intracranial pressure and decreased brain edema [33]. The lactate level and its clearance might be affected by overproduction of lactate from skeletal muscle in PCAS patients with seizures or shivering. If a patient had a seizure, we tried to control seizure activity as soon as possible with antiepileptic drugs. Seizures were myoclonic type in our study, so we supposed that they did not have much impact on lactate clearance. We also tried to control shivering and kept the patient’s Bedside Shivering Assessment Scale <2 using our anti-shivering protocol. In addition, we conducted an analysis to examine a correlation between body mass index and lactate clearance, but the result was not statistically significant (data not shown). The mechanisms regarding the role of lactate and its clearance thus remain to be determined.

### Limitations

There are several limitations to our study. First, this was a retrospective study using a prospective TH registry data. Since the study was not performed on an intention-to-treat basis, there could have been loss of patients, resulting in selection bias. However, every enrolled patient had been treated in accordance with a prospectively designed protocol and patients’ laboratory findings and major information were retrievable except for three patients. Second, this study was a nonexperimental observational study, which can only determine the association and not causal relationships. We could not fully explain the pathophysiology of our findings. Third, our study was conducted with a relatively small population from a single institute, which also may cause potential bias. However, we can assume that patients had relatively homogeneous baseline characteristics because of the specific geographic location and the similarities between the two groups as shown in Table 1. Fourth, in this study, we did not include the medications and methods used during treatment. The doses of epinephrine used during resuscitation and of vasopressor during TH are known to lead to or aggravate hyperlactemia [34], and thus these could be confounders in the analysis. Fifth, we excluded one patient with underlying liver cirrhosis. Owing to his poor liver function, the patient’s basal lactate level before cardiac arrest was already high and we were concerned this might have a deviating effect on our analysis. We could not perform further analysis due to the small number of patients. Further study might be needed for the group of patients with impaired liver function.

## Conclusion

Our study showed that the initial lactate level in OHCA survivors undergoing TH was not associated with neurologic outcome, but that better lactate clearance in the early phase (at 6 hours and 12 hours) showed a significant association with a good neurological outcome.

## Key message

● Lactate clearance at 6 hours and 12 hours in OHCA patients undergoing TH may be useful as a predictor of good neurologic outcome.

## Abbreviations

OHCA: Out-of hospital cardiac arrest; PCAS: Post-cardiac arrest syndrome; ROSC: Return of spontaneous circulation; TH: Therapeutic hypothermia.

## Competing interests

The authors declare that they have no competing interests.

## Authors’ contributions

TRL and MJK participated in the study design, collection of data and drafting of the manuscript. TGS and WCC participated in statistical analysis and helped draft the manuscript. MSS participated in the design of the study and performed statistical analysis and drafted the manuscript. IJJ, KJS, YKJ and JHC participated in the study design and coordination and helped to draft the manuscript. All authors read and approved the final manuscript.
